# Behavioral Interpretation of Willingness to Use Wearable Health Devices in Community Residents: A Cross-Sectional Study

**DOI:** 10.3390/ijerph20043247

**Published:** 2023-02-13

**Authors:** Jiaxin Chen, Ting Li, Hua You, Jingyu Wang, Xueqing Peng, Baoyi Chen

**Affiliations:** 1 School of Nursing, Nanjing Medical University, Nanjing 211166, China; 2 Geriatric Hospital of Nanjing Medical University, Nanjing 210009, China; 3 School of Public Health, Nanjing Medical University, Nanjing 211166, China; 4 Chengdu Center for Disease Control and Prevention, Chengdu 610041, China; 5 MaiGaoQiao Community Health Service Center, Nanjing 210028, China

**Keywords:** wearable health devices (WHDs), theory of planned behavior (TPB), diffusion of innovation theory (DOI), willingness to use, community residents

## Abstract

Wearable health devices (WHDs) have become increasingly advantageous in long-term health monitoring and patient management. However, most people have not yet benefited from such innovative technologies, and the willingness to accept WHDs and their influencing factors are still unclear. Based on two behavioral theories: the theory of planned behavior (TPB) and the diffusion of innovation (DOI), this study aims to explore the influencing factors of willingness to use WHDs in community residents from the perspective of both internal and external factors. A convenience sample of 407 community residents were recruited from three randomly selected Community Health Service Centers (CHSCs) in Nanjing, China, and were investigated with a self-developed questionnaires. The mean score of willingness to use WHDs was 17.00 (range 5–25). In the dimensions of TPB, perceived behavioral control (β = 1.979, *p* < 0.001) was the strongest influencing factor. Subjective norms (β = 1.457, *p* < 0.001) and attitudes (β = 0.651, *p* = 0.016) were also positively associated with willingness. In innovation characteristics of DOI, compatibility (β = 0.889, *p* < 0.001) and observability (β = 0.576, *p* = 0.003) had positive association with the willingness to wear a WHD. This study supports the applicability of the two behavioral theories to interpret the willingness to use WHDs in Chinese community residents. Compared with the innovative features of WHDs, individual cognitive factors were more critical predictors of willingness to use.

## 1. Introduction

### 1.1. Background

With the growing prevalence of chronic diseases and suboptimal health conditions [1], there is a growing need for people to manage their own health [2]. In this context, an increasing number of mobile health (mHealth) technologies are being used widely in disease management and prevention [3], such as Short Messaging Service (SMS), wearable devices and unobserved sensing technologies, and applications running on smartphones [4]. These technologies provide possible tools and technical support for health management [5]. Among them, wearable health devices (WHDs) are particularly suitable for the long-term monitoring of physiological conditions of patients with chronic diseases due to their ease of use and low cost [6,7]. WHDs are emerging mobile medical technologies which can continuously and dynamically monitor physiological health parameters such as heart rate, blood pressure, respiratory rate and blood oxygen saturation in daily life or clinical environment [8]. By transferring them to the medical center, long-term health monitoring, disease prevention and personalized medicine can be realized [9].

A considerable number of WHDs have been developed and validated for monitoring almost all types of important physiological information [5]. Despite the many benefits of WHDs, they are not always sustainably used and accepted by their potential users. A previous study found that WHDs have many potential benefits, specifically aimed at the elderly, but the adoption rate was very low at present [10]. Similarly, a national survey in the United States that recruited 4551 respondents showed that only about 30 percent of adults use WHDs [11]. To ensure that community residents can benefit from the advantages of WHDs to a greater extent, it is important to understand the factors that influence their use willingness. According to previous studies, we found that the factors affecting the acceptance and adoption of WHDs mainly fall into three categories, including: inherent device hardware attributes (such as comfort and safety) [12,13]; user-centered subjective factors (such as perceived ease of use, perceived privacy risk, self-efficacy and attitude) [14,15,16,17,18]; and other engineering design challenges, including privacy, power consumption, and transmission reliability [19,20,21].

Many factors in existing studies have been verified to be related to willingness. Nevertheless, most studies were based on the unified theory of acceptance and use of technology (UTAUT) model [16,22,23] and the technology acceptance model (TAM) [24,25], or focused on improving and extending these models [17,26,27,28]. That means that these studies focused more on technical structure and ignored some other types of determinants [29].

The utilization of WHDs is not only an application of a new technology, but also a proactive health behavior. The willingness to use may be influenced by factors both in features of innovative technologies, and in individual the psychological cognition [1,29,30]. However, few studies have explored the influencing factors of willingness to use WHDs from two perspectives: the characteristics of innovation (e.g., new technologies) itself and the psychological cognition of users. Therefore, the theory of planned behavior (TPB) and diffusion of innovation theory (DOI) models were used as the theoretical basis in this study.

### 1.2. Theoretical Basis

#### 1.2.1. Theory of Planned Behavior

TPB is used to explore the influencing factors of behavior, predict behavioral intention, and try to explain how people decide on health behaviors. It intends to explore the important factors that affect individual behaviors [31]. According to this theory, behavioral intention is the most direct factor affecting actual behavior, and is determined by attitudes (the degree to which performance of the behavior is positively or negatively evaluated), subjective norms (perception of general social pressure from significant others to perform or not to perform a behavior) and perceived behavioral control (peoples’ perceptions of their ability in performing or not performing a given behavior) [32,33]. 

#### 1.2.2. Diffusion of Innovation Theory

DOI could depict the fundamental characteristics of innovation and the adoption curves of people through time [34]. The characteristics of innovation in DOI could help indicate the influencing factors of adoption intention for innovative things, and it has been proven to be a significant predictor of adoption intention [35,36]. The characteristics of innovation include 5 dimensions: (1) relative advantage, (2) compatibility, (3) complexity, (4) trialability, and (5) observability. Due to the high price and cost of WHDs, it is difficult to conduct a free trial among the residents. Therefore, trialability is not selected as a variable in this study, which instead refers to the approach of a similar study [34]. 

TPB has been widely used to explain health-related behaviors. For example, Ye et al. [37] used TPB and other theories to construct a model to analyze the factors of AI (Artificial Intelligence) adoption in healthcare; Deng et al. [38] compared the adoption of mHealth services among middle-aged and elderly Chinese users based on TPB; and Wang et al. [1] integrated TPB with TAM and three patient-centered factors to predict patient acceptance of mHealth platforms [1]. It follows that TPB has a wide range of applications in the adoption of digital health services by individuals, whether using mhealth or WHDs. Therefore, from the perspective of cognitive self-regulation, TPB has the potential to explain an individual’s behavioral intention to adopt WHDs [33]. DOI, as a classical theory, has been widely used in the research field of adoption of new technologies by a certain group. In terms of the application of new technologies in digital health, this theory has been applied in the research on the propagation and diffusion mechanism of new technologies such as wearable activity trackers [34], electronic health [39], wearable devices [40], and mobile medical applications [41]. Since WHDs have only emerged in recent years and remain an innovative technology, DOI has considerable capacity to elucidate the determinant factors in adopting and diffusion of new technology [42]. Based on these two theoretical models, it can not only analyze the influence of internal factors (personal psychological cognition) with regard to willingness, but also explore the impact of external factors (innovation attributes of WHDs).

### 1.3. Research Objectives and Hypotheses

Based on TPB and DOI, this study aims to explore community residents’ willingness to use WHDs. We propose the following study hypothesis: based on TPB theory, H1: A positive attitude will increase the willingness to use WHDs; H2: Subjective norms will increase the willingness to use WHDs; H3: Perceived behavioral control will increase the willingness to use WHDs. Based on the DOI theory, H4: Relative advantage will increase the willingness to use WHDs; H5: Compatibility will increase the willingness to use WHDs; H6: Complexity will reduce the willingness to use WHDs; H7: Observability will increase the willingness to use WHDs. 

Furthermore, by comparing the impact of internal and external factors, the purpose of this study also includes having a fresh look at the key factors, which could provide a theoretical basis and practical strategies for popularizing the use of WHDs.

## 2. Materials and Methods

### 2.1. Data Collection

The study was conducted from February to April 2022 in Nanjing, Jiangsu Province. There are five districts in the main city of Nanjing. We randomly selected three districts, and then selected one community health service center from each district. The residents who visited the community health service center were selected by convenient sampling (the health status of participants was not restricted). The investigators distributed the questionnaires after obtaining informed consent. Before the survey started, a researcher explained the purpose of the study, the concept of WHDs, and details of participation to the respondents. Each questionnaire completed by the participants on a voluntary basis 

### 2.2. Sample Size

There are 14 variables in this study, including six related to demographic information of the participants, seven based on the dimensions of TPB and DOI, and one on the outcome variable. According to the Kendall sample estimation method used for multiple linear analysis, the required sample should be 10–20 times the number of variables [43]. Thus, the minimum sample size for this study was 140 to 280. Ultimately, 435 questionnaires were collected for this study. Excluding invalid samples with incomplete data and the same score for all items, 407 valid questionnaires were obtained, with an effective rate of 93.6%.

### 2.3. Development of the Study Questionnaire

A self-designed questionnaire was used in this study. The first section of the questionnaire involves socio-demographic characteristics, including six variables: gender, age, education level, marital status, working status, and monthly income. The second section consists of 35 questions designed based on TPB and DOI theories to measure seven variables: attitudes, subjective norms, perceived behavioral control, relative advantage, compatibility, complexity, and observability. The items measured for each dimension were mainly adapted from previous studies, which were determined by reviewing the literature related to the application of TPB [37,44,45] and DOI theory [39,41,46,47], and by referring to studies connected with the intention to use WHDs [17,19,48]. The third section is the willingness to use WHDs subscale, which contains five items (e.g., “I try to use WHDs in my daily life”). All items in Section 2 and Section 3 were measured using a 5-point Likert scale, with scores ranging from 1 (strongly disagree) to 5 (strongly agree). The questionnaire does not involve the price of WHDs, so the willingness to use WHDs was investigated without considering the price factor. To test the internal consistency and reliability of the scale, we administered Cronbach’s alpha tests to questions in each dimension of TPB, DOI and willingness to use. Table 1 shows the operational definition and reliability test results of each dimension.

To test whether the questions would be easy to understand or whether revisions were necessary, a pre-survey was conducted among a small sample of individuals from the study site. For the finalized questionnaire, the test results showed good reliability and validity: Cronbach’s alpha for each dimension of TPB, DOI and willingness to use ranged from 0.799 to 0.943; the Kaiser-Meyer-Olkin (KMO) value was 0.960, and Bartlett’s sphericity test was significant (*p* = 0.001). Exploratory factor analysis (EFA) was used to measure seven factors (attitude, subjective norms and perceived behavioral control in TPB, and relative advantage, compatibility, observability, and complexity in DOI). The cumulative explanatory power of the analysis results was 71.06%.

### 2.4. Statistical Analysis

Data were entered simultaneously by two researchers using Epidata3.1. In the descriptive analysis, the continuous variables were represented by the median and interquartile range (non-normal distribution). Categorical data were reported in frequency and proportion. Each dimension was calculated as the arithmetic mean of the total score for all items in this dimension. Univariate linear regression analyses were used to screen out the possible influencing factors. In the multiple linear regressions, the willingness to use WHDs was taken as the dependent variable, and the dimensions of two theories were set as core independent variables. Sociodemographic characteristics were included as covariates (where working status and education were included in the form of categorical variables; age and income were included in the form of continuous type variables). Income was included in the regression analysis after being logarithmized. All regression tests were performed using the Enter method, and the statistical significance was set at *p* < 0.05 (two-sided). Data analyses were performed using SPSS 26.0 for Windows (SPSS Inc., Chicago, IL, USA).

## 3. Results

### 3.1. Participants’ Characteristics

A total of 407 participants with a median age of 58 years were included in this study. The number of females (54.1%) was slightly higher than that of males (45.9%). Among the participants, nearly 70% had a high school education or above. The majority (68.1%) were unemployed and the median monthly income was RMB 4000. (Table 2).

### 3.2. Scores for Each Dimension of Behavioral Theories and Willingness to Use

Table 3 summarizes the descriptive statistics for each dimension based on behavioral theories and on willingness to use. The mean scores of three TPB dimensions were 16.33 (attitudes), 17.00 (subjective norms) and 17.42 (perceived behavioral control), respectively. Meanwhile, the mean scores of four dimensions in DOI were 19.01 (relative advantage), 18.28 (compatibility), 18.23 (complexity) and 15.49 (observability), respectively. The average score of willingness is 17.00. Descriptive statistics for each item are presented in Supplement File S1.

### 3.3. Influencing Factors of Willingness to Use WHDs

#### 3.3.1. Univariate Analysis

The univariate regression analysis (Table 4) showed that the willingness to use WHDs was not only influenced by attitudes, subjective norms, and perceived behavioral control in TPB (*p* < 0.001), but was also related to relative advantage, compatibility, complexity, and observability in DOI (*p* < 0.001). Age, working status, education level and monthly income also had an impact on the willingness to use WHDs (*p* < 0.05).

#### 3.3.2. Multiple Analysis

(Table 5) The results of multiple linear regressions (Model 3) showed that attitudes (β = 0.651, *p* = 0.016), subjective norms (β = 1.457, *p* < 0.001) and perceived behavioral control (β = 1.979, *p* < 0.001) in TPB had positive associations on willingness. The compatibility (β = 0.889, *p* < 0.001) and observability (β = 0.576, *p* =0.003) of DOI also had an impact on the willingness to use WHDs.The adjusted R² of Model 3 was 0.709, indicating a high degree of model fitting.

## 4. Discussion

This study aims to identify predictors for willingness to use WHDs among community residents based on two behavioral theories, TPB and DOI. The results confirm that three dimensions of TPB theory and two dimensions of DOI theory are explanatory factors. 

In this study, with regard to TPB-related variables, the results confirmed that attitudes, subjective norms and perceived behavioral control are all internal influencing factors with regard to the willingness to use WHDs. Perceived behavioral control was the most important determinant of willingness, and this is similar to the findings of some other behavioral intention studies, such as in the prediction of intention to screen for cervical cancer in women [49] and in the research on parental intention to vaccinate their children with the COVID-19 booster vaccine [50]. Furthermore, perceived behavioral control has also been proved to be the major determinant in several studies on m-Health use [44,45,51]. To increase the perception of behavior control, measures should be taken to help target population build confidence in their ability to use WHDs. External support such as providing well-designed training is a promising way to help the target population learn about the design and function of WHDs, which in turn will enhance their willingness [52]. According to previous studies, the price of WHDs also affects individuals’ perceived behavioral control [53,54]. Consequently, the strategy of providing a limited time free trial in the community may be considered [12]. Furthermore, it is suggested that community health workers can help to select and recommend some WHD products which have received good feedback from early users, and to give specific instructions on how to use them. However, in some studies [53,55,56], perceived behavior control had no correlation to willingness, probably because people have started to use smart technology-based products in their daily activities, so they can easily overcome some problems or obstacles during their use and stick to long-term use to monitor their health status. 

Attitudes and subjective norms also had a positive effect on willingness. Consistent with previous studies [28,57], the greater the positive attitude, the higher the willingness to use. Thus, it is crucial for WHDs providers to gain positive comments from community residents by paying more attention to their health needs, and improving the value of WHDs related health services [44]. Of course, for WHDs advocates, the most fundamental thing is to increase the publicity of WHDs effectiveness in health management, which will help create the impression of being “worthy of adoption” among residents [48]. Similarly, subjective norms also positively contribute to willingness, which confirms the findings of several previous studies [15,27,58]. There is no doubt that support from important companions and society can accelerate the psychological acceptance of WHDs, thus motivating more people to use [59]. WHDs have the function of accessories, and the adoption of this innovative technology can reflect a fashionable, healthy, positive and even up-to-date social image [34]. Accordingly, when community residents feel these social images around them, they are more likely to be motivated to use them and thus improve their own social image. In addition, advocates of WHDs should also turn attention to referents, communicating the benefits achievable by the use of WHDs directly to physicians and pharmacists [58]. For those using a device for medical purposes, the encouragement from a health professional can be important for adoption and continued use [59]. Meanwhile, the spread of positive word-of-mouth (e.g., positive ratings) from community members is encouraged in order to amplify subjective norms. [27,30]. 

The results from our study revealed that compatibility and observability in DOI factors were both positive external predictors of willingness. Compatibility refers to how the innovation is aligning with the values, experiences, and needs of potential adopters [60]. Previous studies have confirmed that compatibility is a determining factor in the acceptance of new technologies [18,42]. When using WHDs, individuals will care about whether the features of WHDs, such as health monitoring and exercise reminders, are matched with their health needs. Similarly, individuals with prior experience using digital device technology for health-related purposes will be more compatible with the use of WHDs [11]. From this perspective, the suggestions that can be given to manufacturers of WHDs are to further improve the functionality and upgrade their services based on the existing ones [61]. First, the scope of functions should match the health needs of users and develop accordingly; second, unnecessary interactions with the WHDs should be avoided, such as frequent charging and excessive responding to alarms [59]. Finally, WHDs should be easy to wear, comfortable to wear at night, and as waterproof as possible [12,24]. They should cause minimal disruption to the user’s daily life and work environment, thus increasing compatibility. The positive relationship of observability on willingness to use is also one of the results consistent with other studies [28,34,47]. Observability is an important way to increase the user’s perception of the experience. The observable information should be able to be recognized and understood by users. If potential users cannot understand the meanings of the indicators displayed on the devices, they will lose interest in using them, largely because the information cannot provide them with valuable experiences. Hence, the choice of more understandable health information in the form of output expression, including the presentation of dynamic changes of some indicators, should be considered as an important direction to improve observability. In addition, because WHDs need to be carried around, a beautiful or stylish looking product appearance is more likely to meet the desired social image of community residents [18,28]

Finally, according to the results of this study, perceived behavioral control exerted the greatest influence on willingness to use (β = 1.979), followed by subjective norms (β = 1.457). The finding indicated that internal factors (individual’s cognitive factors) appeared to be more important to affect willingness to use WHDs than external factors (characteristics of innovations). Previous studies on m-Health have also confirmed the significance of individual cognitive factors on adoption intention [30,62]. Psychological perception is usually a sensitive and active factor. Compared with the progress of technology, changing the rational cognition of individuals is obviously easier to implement and more likely to be achieved in the short term. Therefore, great emphasis should be placed on the internal cognitive factors among potential users in WHD diffusion.

However, this study still has several limitations: First, this study used a convenience sampling method and limited the participants to residents visiting community health centers, thus restraining the extrapolation of the conclusions to the general population. Second, the cross-sectional design made it difficult to establish causal relationships. Future studies could be designed longitudinally to better understand how individuals’ willingness to use changes over time. In addition, quantitative results could be combined with qualitative results to provide a more comprehensive insight. Third, this study only investigated willingness to use, but willingness to use is not the same as actual use. Future studies could consider the actual behavior of WHD use and explore whether there is a gap between willingness and actual use.

## 5. Conclusions

On the basis of TPB and DOI theory, this study explored the determinants of willingness to use WHDs. The results corroborated that attitudes, subjective norms, and perceived behavioral control are internal predictors of willingness to use. From the perspective of external factors, compatibility and observability also have an association with willingness. However, psychological perceptions (internal factors) are relatively more critical determinants. Despite some limitations, the results of this study support the applicability of two behavioral theories, TPB and DOI, in predicting the willingness to use WHDs. The results of this study are expected to be utilized by future researchers and practitioners to improve the acceptability of WHDs in healthcare.

## Figures and Tables

**Table 1:** Results of Reliability Test of Theoretical Dimensions and Willingness.

Dimensions		Operational Definition	Number of Items	Example	Cronbach’s Alpha
TPB	Attitudes	Attitude is the degree to which an individual’s use of WHDs is evaluated positively or negatively (Attitude is set as positive evaluation in this study)	5	I think using WHDs is the right choice	0.799
	Subjective norms	Subjective norms are general social pressures that individuals feel from significant others to use WHDs	5	If my family and relatives use WHDs, I will use it too	0.837
	Perceived behavioral control	Perceived behavioral control refers to an individual’s perception of their ability to use WHDs	5	I was able to overcome problems in the use of WHDs	0.903
DOI	Relative advantage	Relative advantage reflects the extent to which individuals find the use of WHDs useful	5	I think using WHDs helps patients reduce healthcare costs	0.912
	Compatibility	Compatibility refers to whether the WHDs are compatible with an individual’s existing values, experiences and needs	5	Using WHDs is compatible with my existing electronic devices (smartphone, etc.)	0.902
	Complexity	Complexity refers to how easily WHDs can be understood or used	5	I can easily use WHDs to monitor my health	0.943
	Observability	Observability is whether the benefits of WHDs are easily observable and visible.	5	I can instantly read my health measurement information from WHDs.	0.915
Willingness to use		The subjective odds of the personal use of WHDs.	5	I try to use WHDs in my daily life	0.912

**Table 2:** Demographic characteristics of the participants (N = 407).

Characteristic		*N* (%), M (IQR)
Age (years)		58 (39–66)
Gender		
	Male	187 (45.9)
	Female	220 (54.1)
Education level		
	Junior school and below	129 (31.7)
	High school	130 (32.0)
	College and above	148 (36.3)
Marital status		
	Married	322 (79.1)
	Unmarried, divorced, widowed	85 (20.9)
Working status ^a^		
	Employed	130 (31.9)
	Unemployed	277 (68.1)
Monthly income (RMB) ^b^		4000 (2000–6000)

^a^ Unemployed refers to retired, school students, jobless or unemployed, freelance, farmers and self-employed; ^b^ 1 RMB = USD 0.1403; 4000 RMB is approximately equal to 561 USD.

**Table 3:** Descriptive statistics of variables.

Variables	Score Range	Min	Max	Mean ± SD	95%CI
TPB					
Attitudes (positive evaluation)	5–25	5	25	16.33 ± 3.55	(15.98, 16.67)
Subjective norms	5–25	6	25	17.00 ± 3.78	(16.63, 17.37)
Perceived behavioral control	5–25	7	25	17.42 ± 3.97	(17.04, 17.81)
DOI					
Relative advantage	5–25	5	25	19.01 ± 3.98	(18.63, 19.40)
Compatibility	5–25	7	25	18.28 ± 3.86	(17.90, 18.66)
Complexity	5–25	7	25	18.23 ± 4.54	(17.80, 18.68)
Observability	5–25	5	25	15.49 ± 4.42	(15.06, 15.92)
Willingness to use	5–25	7	25	17.00 ± 4.18	(16.59, 17.40)

**Table 4:** Univariate analysis of willingness to use wearable health devices.

Variables	β (95% CI)	*p* Value
Demographic characteristics		
Gender	−0.009 (−0.827,−0.809)	0.983
Marital status	−0.898 (−1.897,0.100)	0.078
Age	−0.038 (−0.061,−0.015)	0.001
Working status	1.296 (0.431,2.160)	0.003
Education level	0.975 (0.489,1.460)	<0.001
Monthly income	0.142 (0.009,0.276)	0.037
TPB		
Attitudes (positive evaluation)	3.924 (3.497,4.361)	<0.001
Subjective norms	4.038 (3.669,4.406)	<0.001
Perceived behavioral control	4.100 (3.779,4.421)	<0.001
DOI		
Relative advantage	2.707 (2.268,3.146)	<0.001
Compatibility	3.617 (3.225,4.010)	<0.001
Complexity	2.839 (2.485,3.193)	<0.001
Observability	2.914 (2.551,3.277)	<0.001

**Table 5:** Multiple analysis of willingness to use wearable health devices.

Variables	Model 1 ^a^		Model 2 ^a^		Model 3 ^a^	
	Β (95% CI)	*p* Value	Β (95% CI)	*p* Value	Β (95% CI)	*p* Value
TPB						
Attitudes (positive evaluation)	1.244 (0.779–1.708)	<0.001			0.651 (0.122–1.180)	0.016
Subjective norms	1.694 (1.220–2.169)	<0.001			1.457 (0.980–1.935)	<0.001
Perceived behavioral control	2.171 (1.704–2.637)	<0.001			1.979 (1.455–2.502)	<0.001
DOI						
Relative advantage			0.543 (0.090–0.955)	0.019	0.086 (−0.299–0.470)	0.662
Complexity			0.803 (0.383–1.223)	<0.001	−0363 (−0.756–0.030)	0.070
Compatibility			1.810 (1.269–2.352)	<0.001	0.889 (0.420–1.358)	<0.001
Observability			1.409 (1.020–1.798)	<0.001	0.576 (0.203–0.948)	0.003

^a^ Model1 included the dimensions of TPB as independent variables; Model 2 included the dimensions of DOI as independent variables; Model 3 included all the dimensions of TPB and DOI as independent variables; Model 1–3 were included the demographic characteristics as the control variables.

## Data Availability

The dataset supporting the conclusions of this article is available from the corresponding author on reasonable request.

## Supplementary Materials

The following supporting information can be downloaded at: https://www.mdpi.com/article/10.3390/ijerph20043247/s1, File S1: Descriptive statistics for each item.
