# Supplementary material for: Behavioral Interpretation of Willingness to Use Wearable Health Devices in Community Residents: A Cross-Sectional Study
Source: Int J Environ Res Public Health. 2023 Feb 13;20(4):3247. doi: 10.3390/ijerph20043247 (PMC9960868; doi:10.3390/ijerph20043247)
Supplement: Supplementary file 1 [file ijerph-20-03247-s001.zip › ijerph-2098767-supplementary.pdf]

Supplementary File S1.

Descriptive statistics for each item

Dimensions	Code	Score（M±SD）
Attitudes	ATT1	3.49±0.90
	ATT2	3.61±0.91
	ATT3	3.44±0.91
	ATT4	2.91±1.00
	ATT5	2.88±1.05
Subjective norms	SN1	3.25±0.98
	SN2	3.62±0.95
	SN3	2.91±0.95
	SN4	3.50±1.01
	SN5	3.72±0.98
Perceived behavioral control	PBC1	3.44±0.92
	PBC2	3.53±0.91
	PBC3	3.29±1.00
	PBC4	3.54±0.89
	PBC5	3.63±0.97
Relative advantage	RA1	3.44±0.93
	RA2	3.90±0.94
	RA3	3.81±0.94
	RA4	3.94±0.91
	RA5	3.92±0.92
Compatibility	CO1	3.71±0.91
	CO2	3.52±0.94
	CO3	3.94±0.87
	CO4	3.64±0.95
	CO5	3.47±0.89
Complexity	CM1	3.64±1.00
	CM2	3.61±0.98
	CM3	3.61±0.99
	CM4	3.71±1.01
	CM5	3.68±1.02
Observability	OB1	3.38±0.94
	OB2	3.26±0.99
	OB3	2.96±1.06
	OB4	2.96±1.06
	OB5	2.95±1.08
Willingness to use	WTU1	3.23±1.02
	WTU2	3.16±0.99
	WTU3	3.43±0.97

	WTU4	3.34±0.98
	WTU5	3.84±0.91
